# Accuracy and Diversity of Wearable Device–Based Gait Speed Measurement Among Older Men: Observational Study

**DOI:** 10.2196/29884

**Published:** 2021-10-11

**Authors:** Min-gu Kang, Seong-Ji Kang, Hye-Kang Roh, Hwa-Young Jung, Sun‑wook Kim, Jung-Yeon Choi, Kwang-il Kim

**Affiliations:** 1 Department of Internal Medicine Chonnam National University Bitgoeul Hospital Gwangju Republic of Korea; 2 Graduate School of Health Science and Management Yonsei University Seoul Republic of Korea; 3 WELT Corp, Ltd, Seoul, Korea Seoul Republic of Korea; 4 Department of Internal Medicine Seoul National University Bundang Hospital Seongnam Republic of Korea; 5 Department of Internal Medicine Seoul National University College of Medicine Seoul Republic of Korea

**Keywords:** gait speed, sarcopenia, skeletal muscle mass, wearable device

## Abstract

**Background:**

Gait speed measurements are widely used in clinical practice, as slow gait is a major predictor of frailty and a diagnostic criterion for sarcopenia. With the development of wearable devices, it is possible to estimate the gait speed in daily life by simply wearing the device.

**Objective:**

This study aims to accurately determine the characteristics of daily life gait speed and analyze their association with sarcopenia.

**Methods:**

We invited community-dwelling men aged >50 years who had visited the outpatient clinic at a tertiary university hospital to participate in the study. Daily life gait speed was assessed using a wearable smart belt (WELT) for a period of 4 weeks. Data from participants who wore the smart belt for at least 10 days during this period were included. After 4 weeks, data from a survey about medical and social history, *usual gait speed* measurements, handgrip strength measurements, and dual-energy x-ray absorptiometry were analyzed.

**Results:**

A total of 217,578 daily life gait speed measurements from 106 participants (mean age 71.1, SD 7.6 years) were analyzed. The mean daily life gait speed was 1.23 (SD 0.26) m/s. The daily life gait speed of the participants varied according to the time of the day and day of the week. Daily life gait speed significantly slowed down with age (*P*<.001). Participants with sarcopenia had significantly lower mean daily life gait speed (mean 1.12, SD 0.11 m/s) than participants without sarcopenia (mean 1.23, SD 0.08 m/s; *P*<.001). Analysis of factors related to mean daily life gait speed showed that age and skeletal muscle mass of the lower limbs were significantly associated characteristics.

**Conclusions:**

More diverse and accurate information about gait speed can be obtained by measuring daily life gait speed using a wearable device over an appropriate period, compared with one-time measurements performed in a laboratory setting. Importantly, in addition to age, daily life gait speed is significantly associated with skeletal muscle mass of the lower limbs.

## Introduction

### Background

Globally, populations are aging, including in Korea, where the population is aging rapidly because of extended life expectancy [1] and a low fertility rate. In 2017, more than 14% of the total population in Korea was aged ≥65 years.

Frailty, as a reflection of decreased physiological reserve, is closely associated with increased biological age [2], concurrent medical conditions, morbidity, and decreased survival in older individuals [3]. Frailty assessments are clinically useful for determining the heterogeneous health status of older individuals [4]. Sarcopenia, defined as low muscle mass and low muscle strength, is a key characteristic of frailty [5-7]. Recently, the treatment of sarcopenia has become a key strategy for preventing and overcoming frailty [8].

Slow gait speed is a major feature of frailty [9] and a diagnostic criterion for sarcopenia [6,7]; therefore, gait speed measurement is widely used to assess frailty. However, gait speed measurement methods are not completely standardized, and the term *usual gait speed* refers to a measure that has been commonly used as part of this fast, safe, and inexpensive assessment [10,11]. *Usual gait speed* is calculated by instructing the individual to walk a certain distance at their usual pace in the laboratory and then measuring the time it takes to cover that distance.

Currently, technology is advancing at a rapid pace in the area of wearable devices, which can be used to measure a variety of attributes. Wearable sensors allow frequent and continuous measurements of body functions, including various physical activities, such as walking, running, and biking [12]. With the availability of these wearable devices, it is possible to measure and track daily life gait speed in the real world without significant additional effort by asking the patient to wear the device [13,14]. Moreover, it is questionable whether the *usual gait speed* measured in the laboratory can represent daily gait speed in an individual’s real world [15]. Few studies have compared *usual gait speed* and real-world gait speed in analyses of the associations between muscle mass or muscle strength and gait speed.

### Objectives

The primary aim of this study was to identify the characteristics of daily life gait speed in community-dwelling older male adults and to analyze the association of these characteristics with sarcopenia. The secondary objective was to compare the results of daily life gait speed obtained using a wearable device with *usual gait speed* measured in the laboratory by analyzing the associations of muscle mass and muscle strength with gait speed.

## Methods

### Study Population

This observational study was conducted at Seoul National University Bundang Hospital. Men aged >50 years who could walk unassisted were recruited consecutively from November 16, 2018, to April 12, 2019.

### Study Protocol

The WELT (WELT Corp, Ltd) is a belt-type wearable device that uses a triaxial accelerometer to continuously measure the wearer’s gait speed while walking. The smart belt measures the walking speed using the step interval time and stride length. An algorithm for detecting and analyzing peaks is used to identify the steps. Two consecutive peaks are detected as steps occur, and the step interval time between the peaks is measured in 0.1-second increments. There are several ways to estimate stride length, and WELT adopts a method that uses a sex-based constant and height [16,17]. As a result, it is possible to continuously measure gait speed that changes with the step interval time.

Participants were asked to wear the WELT for 4 weeks as they went about their daily lives, and their daily life gait speed was recorded. After 4 weeks, a survey that included questions about social history and past medical history was administered, *usual gait speed* and handgrip strength measurements were obtained, and a dual-energy x-ray absorptiometry (DEXA) was performed.

*Usual gait speed* was calculated for each participant using distance in meters and time in seconds. A marked walkway and an automated laser-gated chronometer attached to the wall were used to calculate gait speed [18]. The walkway consisted of a 1-m acceleration phase, 4.5-m timed section, and a 1-m deceleration phase. The chronometer started and stopped automatically as each participant crossed into and out of the timed section. The participants were instructed to walk at their usual pace. Handgrip strength was measured using a Jamar Plus digital hand dynamometer (Patterson Medical). Participants were seated in a chair with their shoulder adducted and neutrally rotated, their elbow flexed to 90°, and their forearm and wrist neutrally positioned. A total of three consecutive measurements of the dominant hand were taken with a brief rest between measurements, and the average scores were obtained and recorded in kilograms. DEXA was used to measure the limb skeletal muscle mass of the participants. It is very useful for body composition analysis because fat mass, nonbone lean mass, and bone mineral content can be differentiated at both the regional and whole-body levels [19]. A Horizon W machine (Hologic Inc) was used to measure appendicular skeletal muscle mass [20]. Before the DEXA scan, participants were asked to remove all metal objects and change into hospital gowns. Scanning was performed by an imaging technician in the laboratory. The scan time was approximately 10 minutes. Appendicular skeletal muscle mass was calculated as the sum of the muscle mass of both arms and legs.

Sarcopenia was defined based on the diagnostic criteria of the Asian Working Group for Sarcopenia [6]. Low muscle strength was defined as a handgrip measurement of <28 kg. Low physical performance was defined as a *usual gait speed* of <1.0 m/s. Sarcopenia was diagnosed when the individual demonstrated a low appendicular skeletal muscle mass and low muscle strength or low physical performance.

Daily life gait speed was analyzed using data from participants who wore the wearable device for at least 10 days during the 4-week study period. The characteristics of daily life gait speed in the real world were identified, and the association between daily life gait speed and sarcopenia was analyzed. In addition, *usual gait speed* and daily life gait speed were compared by an analysis of the association between muscle mass or muscle strength and gait speed.

### Statistical Analysis

All statistical analyses were performed using SPSS version 21.0 (IBM Corporation). Continuous variables were expressed as mean (SD) and were compared using either a two-tailed unpaired *t* test or one-way analysis of variance. Discrete variables were expressed as counts and percentages, and the proportions were compared using the chi-square test or Fisher exact test. We used correlation analysis and linear regression analysis to identify factors related to daily life gait speed. All statistical analyses were two tailed, and *P*<.05 were considered statistically significant.

### Ethical Standards

This study was approved by the institutional review board of Seoul National University Bundang Hospital (Institutional Review Board No. B-1808/486-002). All participants provided written informed consent before participation. The study was performed in accordance with the principles of the Declaration of Helsinki.

## Results

### Characteristics of Study Participants

**Figure 1:**
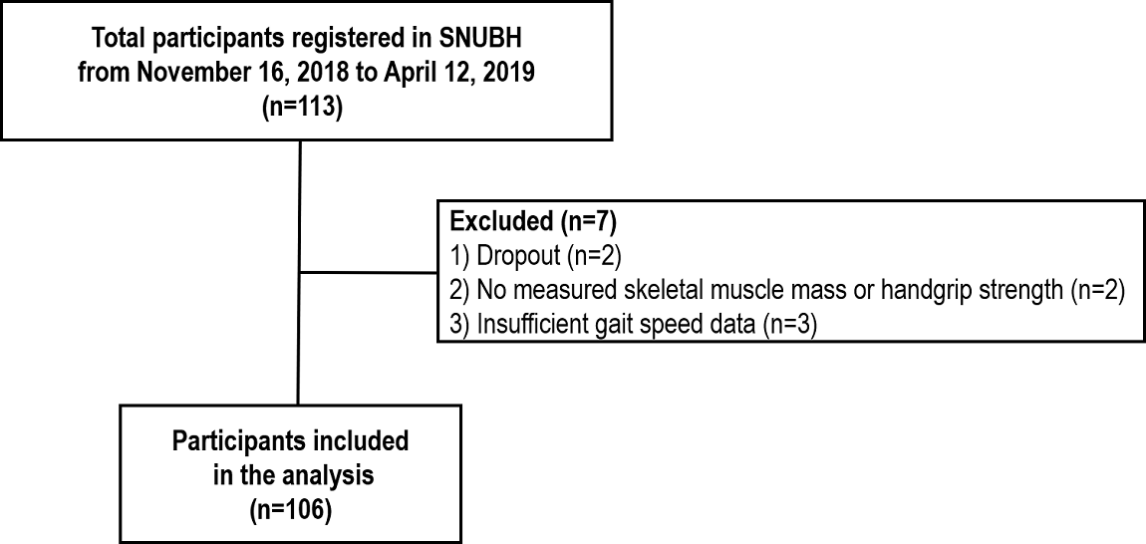
Flow diagram of the study population. SNUBH: Seoul National University Bundang Hospital.

A total of 217,578 daily life gait speed measurements were analyzed from 106 participants who completed the study (Figure 1). The average age of the participants was 71.1 (SD 7.6; range 52-90) years, and the average number of gait speed measurements per participant was 2052.6 (SD 1022.3; range 618-4783). The mean daily life gait speed of the participants was 1.23 (SD 0.26; range 0.94-1.39) m/s, and the average walking time per day was 88.0 (SD 40.2; range 30.5-176.5) minutes.

### Characteristics of Daily Life Gait Speed Measured Using a Wearable Device

**Figure 2:**
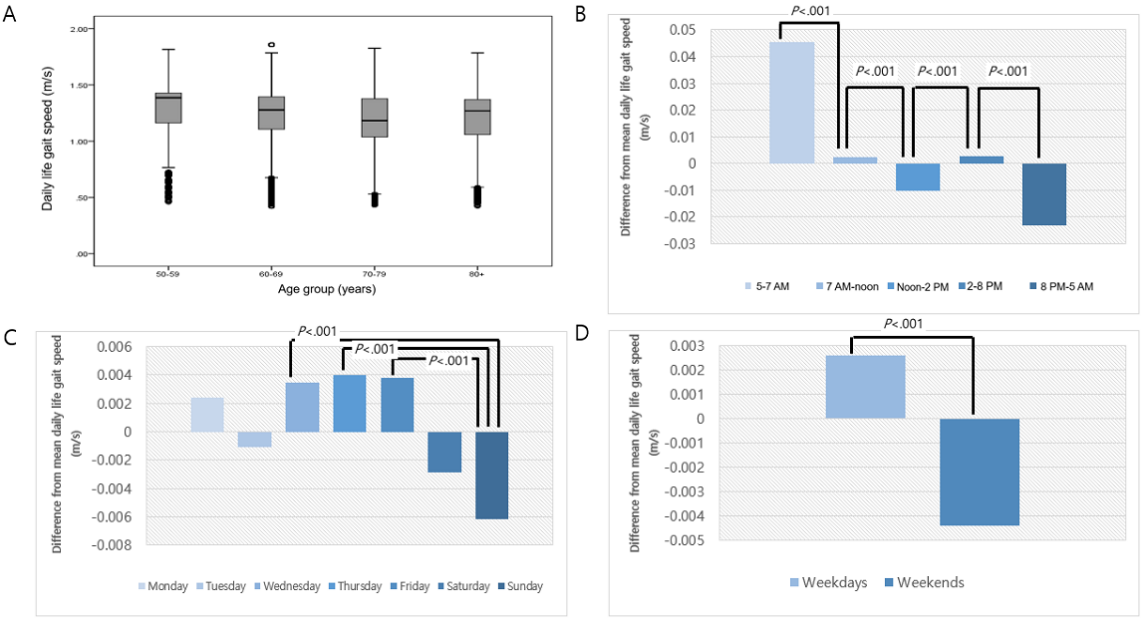
The characteristics of daily life gait speed measured using a wearable device. Comparison of daily life gait speed (A) by age group; (B) by time of day; (C) by day of the week; (D) between weekdays and weekends.

The characteristics of daily life gait speed were presented in Figure 2. The daily life gait speed was significantly lower in older individuals (*P*<.001). Participants walked the fastest between 5 AM and 7 AM and the slowest at night (*P*<.001). There was a statistically significant difference in daily life gait speed according to the day of the week (*P*<.001), and weekday gait speed (mean 1.23, SD 0.26 m/s) was significantly faster than weekend gait speed (mean 1.22, SD 0.26 m/s; *P*<.001). Walking time per day differed significantly on weekdays (mean 93. 4, SD 47.0 minutes) and weekends (mean 74.5, SD 41.6 minutes; *P*<.001).

**Figure 3:**
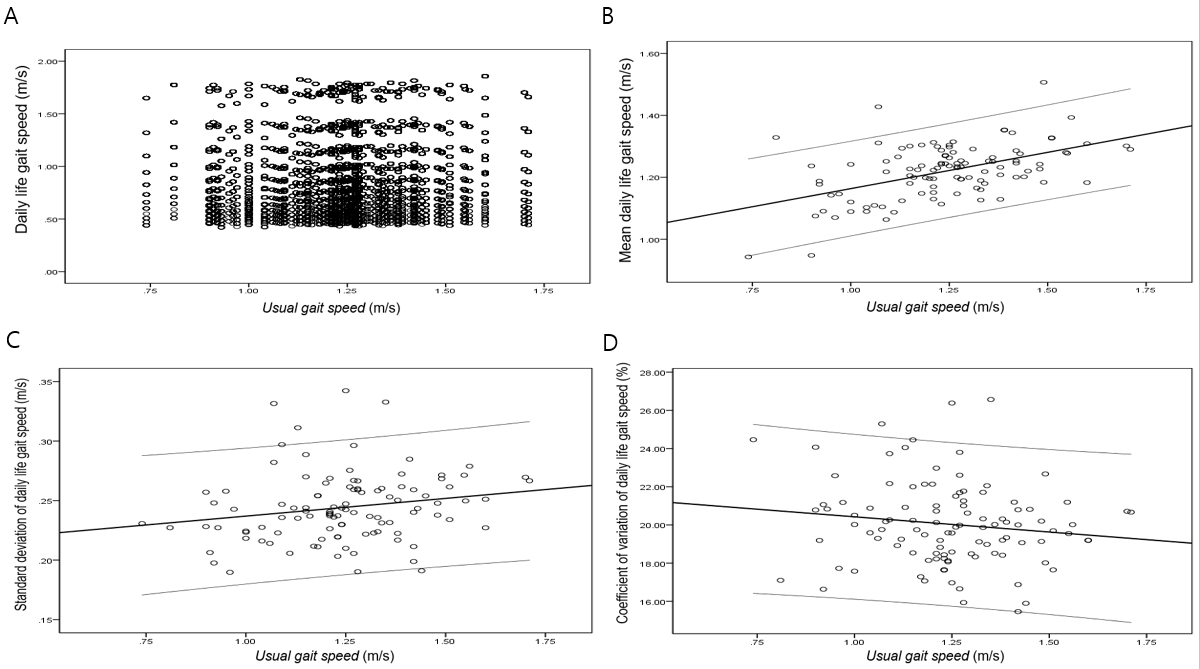
Scatterplot of daily life gait speed and usual gait speed. (A) Scatterplot of daily life gait speed and usual gait speed (Pearson correlation coefficient = 0.155; *P*<.001). (B) Scatterplot of mean daily life gait speed and usual gait speed (Pearson correlation coefficient = 0.504; *P*<.001). (C) Scatterplot of SD of daily life gait speed and usual gait speed (Pearson correlation coefficient = 0.195; *P*=.046). (D) Scatterplot of coefficient of variation of daily life gait speed and usual gait speed (Pearson correlation coefficient = −0.139; *P*=.16).

To compare daily life gait speed with *usual gait speed*, the daily life gait speed for each participant was calculated as a percentile compared with their own *usual gait speed*. The participants’ daily life gait speed varied widely. The corresponding percentiles ranged from 30.3 percentile to 181.0 percentile, with a median value of 102.9 percentile. In their daily lives, a walking speed >25% faster than their own *usual gait speed* was observed in 13.46% (29,279/217,578) of the total number of measurements, and a walking speed >25% slower than their own *usual gait speed* was observed in 14.58% (31,728/217,578) of the total number of measurements. In the analysis of the correlation between daily life gait speed and *usual gait speed*, only a negligible correlation was confirmed (Pearson *r*=0.155; *P*<.001; Figure 3). However, in the analysis of the correlation between mean daily life gait speed and *usual gait speed*, a moderate positive correlation was confirmed (Pearson *r*=0.504; *P*<.001; Figure 3). In the analysis of the correlation between the SD of daily life gait speed and *usual gait speed*, only a negligible correlation was confirmed (Pearson *r*=0.195; *P*=.046; Figure 3). In addition, no significant correlation was found between the coefficient of variation of daily life gait speed and *usual gait speed* (Pearson *r*=−0.139; *P*=.16; Figure 3).

### Comparison Between Study Participants With and Without Sarcopenia

Participants with low muscle strength (handgrip strength <28 kg) had significantly lower daily life gait speed than those with normal muscle strength (mean 1.15, SD 0.25 m/s vs mean 1.23, SD 0.26 m/s; *P*<.001). There were also significant differences in daily life gait speed between participants with low muscle mass (appendicular skeletal muscle mass <7.0 kg/m^2^) and those with normal muscle mass (mean 1.22, SD 0.26 m/s vs mean 1.25, SD 0.26 m/s; *P*<.001).

A total of 13 participants (13/106, 12.3%) were diagnosed with sarcopenia based on the diagnostic criteria of the Asian Working Group for Sarcopenia. Participants with sarcopenia (mean 76.0, SD 6.2 years) were older than participants without sarcopenia (mean 70.5, SD 7.6 years; *P*=.01) and had significantly lower mean daily life gait speed than normal participants (mean 1.12, SD 0.11 m/s vs mean 1.23, SD 0.08 m/s; *P*<.001). Sarcopenic participants had significantly lower handgrip strength and lower appendicular skeletal muscle mass. In addition, among participants with sarcopenia, the proportion of patients who had angina and were current smokers was significantly higher (Table 1).

**Table 1:** Comparison between study participants with and without sarcopenia.

Characteristics		Total (N=106)	Sarcopenia (n=13)	Normal (n=93)	*P* value	
**Age (years)**						.01
	Mean (SD)	71.1 (7.6)	76.0 (6.2)	70.5 (7.6)		
	Range	52-90	67-88	52-90		
**BMI (kg/m^2^)**						.54
	Mean (SD)	24.6 (2.5)	25.0 (2.5)	24.5 (2.5)		
	Range	17.0-30.8	20.7-29.2	17.0-30.8		
**Education period (years)**						.38
	Mean (SD)	14.3 (3.2)	13.5 (3.3)	14.4 (3.2)		
	Range	6-20	8-20	6-20		
**Job status**						.79
	Incumbent	44 (41.5)	5 (38.5)	39 (41.9)		
	Retired	61 (57.5)	8 (61.5)	53 (57)		
Hypertension, n (%)		67 (63.2)	11 (84.6)	56 (60.2)	.13	
Diabetes mellitus, n (%)		28 (26.4)	3 (23.1)	25 (26.9)	.99	
Angina, n (%)		22 (20.8)	6 (46.2)	16 (17.2)	.03	
Arthritis, n (%)		18 (17)	2 (15.4)	16 (17.2)	.99	
**Smoking, n (%)**						.004
	Current smoker	16 (15.1)	6 (46.2)	10 (10.8)		
	Ex-smoker	76 (71.7)	6 (46.2)	70 (75.3)		
	Never smoker	14 (13.2)	1 (7.7)	13 (14)		
**Usual gait speed (m/s)**						<.001
	Mean (SD)	1.24 (0.19)	0.97 (0.12)	1.28 (0.16)		
	Range	0.74-1.71	0.74-1.20	0.91-1.71		
**Daily life gait speed (m/s)**						<.001
	Mean (SD)	1.22 (0.09)	1.12 (0.11)	1.23 (0.08)		
	Range	0.94-1.39	0.94-1.33	1.06-1.39		
**Handgrip strength^a^ (kg)**						<.001
	Mean (SD)	36.0 (6.6)	27.4 (5.4)	37.2 (5.8)		
	Range	22.1-50.3	22.1-38.0	24.5-50.3		
**Appendicular skeletal muscle mass/height^2^ (kg/m^2^)**						.003
	Mean (SD)	6.74 (0.74)	6.39 (0.34)	6.79 (0.77)		
	Range	5.21-8.87	5.68-6.97	5.21-8.87		

^a^Average grip strength measured three times in the dominant hand.

Participants with sarcopenia walked faster from 5 AM to 7 AM than at other times of the day (*P*<.001); however, there were no significant differences between weekday and weekend gait speeds (*P*=.64). Participants with sarcopenia (mean 78.2, SD 45.4 minutes) had significantly less walking time per day than participants without sarcopenia (mean 89.3, SD 46.3 minutes; *P*=.03). In addition, there was no significant difference in walking time per day between weekdays (mean 82.4, SD 46.3 minutes) and weekends (mean 67.8, SD 42.1 minutes) in the participants with sarcopenia (*P*=.17). In the analysis of the correlation between daily walking time and sarcopenia-related factors, it was observed that grip strength, skeletal muscle mass of the lower limbs, and *usual gait speed* had a negligible correlation with walking time (walking time and grip strength: Pearson *r*=0.111; *P*=.003; walking time and skeletal muscle mass of the lower limbs: Pearson *r*= 0.152; *P*<.001; and walking time and *usual gait speed*: Pearson *r*=0.112; *P*=.002). Analysis of the correlation between daily walking time and mean daily life gait speed revealed a low positive correlation (Pearson *r*=0.315; *P*<.001).

There was no significant difference between the mean daily life gait speed (mean 1.12, SD 0.11 m/s) and the median value of daily life gait speed (mean 1.11, SD 0.12 m/s; *P*=.45) in participants with sarcopenia, but those without sarcopenia had median values (mean 1.27, SD 0.13 m/s) significantly higher than the mean values (mean 1.23, SD 0.08 m/s; *P*<.001). There was no significant difference in the SD of daily life gait speed in participants with sarcopenia (mean 0.23, SD 0.02 m/s) and those without sarcopenia (mean 0.25, SD 0.03 m/s; *P*=.06). There was also no significant difference in the coefficient of variation in daily life gait speed between participants with sarcopenia (mean 20.58%, SD 2.37%) and those without sarcopenia (mean 19.96%, SD 2.13%; *P*=.34).

### Factors Associated With Mean Daily Life Gait Speed

The correlation analysis of the potential factors associated with mean daily life gait speed showed that age, height, and skeletal muscle mass of the lower limbs were significantly correlated with mean daily life gait speed (Table 2). In a linear regression analysis with correlated factors, age and lower skeletal muscle mass were significantly associated with mean daily life gait speed. Participants who were younger and had more skeletal muscle mass in their lower limbs walked faster (Table 3).

**Table 2:** Correlation analysis (Pearson *r* and one/two-tailed *P* values) with mean daily life gait speed.^a^

Variable			Mean daily life gait speed		Age		Height		Body weight		BMI		Skeletal muscle mass of the lower limbs
**Mean daily life gait speed**													
	*r*	1		−0.357		0.333		0.108		−0.074		0.383	
	*P* value	—^b^		<.001		<.001		.27		.45		<.001	
**Age**													
	*r*	−0.357		1		−0.274		−0.237		−0.073		−0.338	
	*P* value	<.001		—		.004		.01		.46		<.001	
**Height**													
	*r*	0.333		−0.274		1		0.466		−0.062		0.523	
	*P* value	<.001		.004		—		<.001		.53		<.001	
**Body weight**													
	*r*	0.108		−0.237		0.466		1		0.824		0.797	
	*P* value	.27		.01		<.001		—		<.001		<.001	
**BMI**													
	*r*	−0.074		−0.073		−0.062		0.824		1		0.596	
	*P* value	.45		.46		.53		<.001		—		<.001	
**Skeletal muscle mass of the lower limbs**													
	*r*	0.383		−0.338		0.523		0.797		0.596		1	
	*P* value	<.001		<.001		<.001		<.001		<.001		—	

^a^The correlation is significant at a level of .001 (one/two-tailed).

^b^Not applicable.

**Table 3:** Linear regression analysis.^a^

Independent variable	B	β	*t* statistic	*P* value	Variance inflation factor
Constant	0.837	N/A^b^	N/A	N/A	N/A
Age (years)	−0.003	−.240	−2.566	.01	1.146
Height (m)	0.259	.150	1.454	.15	1.397
Skeletal muscle mass of the lower limbs (kg)	0.011	.223	2.116	.04	1.459

^a^Dependent variable is mean daily life gait speed.

^b^N/A: not applicable.

### Comparison Between Daily Life Gait Speed and Usual Gait Speed in the Analysis of Associations With Muscle Mass and Muscle Strength

A correlation analysis of gait speed, handgrip strength, and appendicular skeletal muscle mass showed that mean daily life gait speed was significantly and positively correlated with handgrip strength (Pearson *r*=0.380; *P*<.001) and appendicular skeletal muscle mass (Pearson *r*=0.355; *P*<.001). *Usual gait speed* had a significant positive correlation with handgrip strength (Pearson *r*=0.501; *P*<.001) but was less correlated with muscle mass (Pearson *r*=0.227; *P*=.02).

## Discussion

### Principal Findings

In this study, we showed that daily life gait speeds varied depending on the time of day and day of the week. Daily life gait speed showed greater variability than *usual gait speed*. The mean daily life gait speed was lower in participants with sarcopenia. When analyzing factors related to daily life gait speed, age and skeletal muscle mass of the lower limbs were significantly associated with mean daily life gait speed. The mean daily life gait speed was significantly and positively correlated with handgrip strength and appendicular skeletal muscle mass.

Walking speed is a valid and reliable measure for assessing the functional status of older adults [21], and identification of a slow walking speed is a simple approach to the diagnosis of frailty [22]. Gait speed at an individual’s usual pace is known to be associated with survival in older adults [23] and has been widely used as a tool to predict adverse outcomes in community-dwelling older individuals, including falls, disability, institutionalization, and mortality [9,24]. However, one study demonstrated that *usual gait speed* does not represent daily life gait speed because it is not strongly related to daily gait speed [15]. Assessment of daily life gait speed showed that the men in our study walked significantly faster in the morning than during the rest of the day, and men with sarcopenia also walked faster in the morning. People may walk faster in the early morning because they usually have to go to work or attend social activities at a specified time. Participants in our study also walked faster on weekdays than on weekends. However, this difference was not significant in individuals with sarcopenia. This may be related to a decrease in the daily life gait speed of participants with sarcopenia, especially a decrease in the use of a fast gait speed. This is supported by the fact that men without sarcopenia often walked faster than their mean daily life gait speed, whereas participants with sarcopenia did not. As gait speed varied depending on the time of day and day of the week and the distribution of gait speed was very diverse, it would be difficult to accurately reflect the actual gait speed of individuals using one or two *usual gait speed* measurements obtained in the clinical setting. In fact, we found that the Pearson correlation coefficient between daily life gait speed and *usual gait speed* was negligible [25]. In addition, although the direct correlation between walking time and sarcopenia-related factors is not clear, people who spend more time walking tend to walk faster in their daily lives. An increase in walking-related exercise may be associated with a decrease in sarcopenia [26].

In this study, the mean value was used to represent the daily life gait speed to identify factors related to gait speed and to analyze the associations between gait speed and sarcopenia. Age and skeletal muscle mass of the lower limbs were significantly associated with the mean daily life gait speed. Reduced walking speed is associated with increasing age [27,28]. A decrease in the muscle mass of the lower limbs is also associated with a decrease in gait speed [29].

The participants in our study who had sarcopenia walked significantly more slowly in their daily lives than those without sarcopenia. This result was expected considering that patients with sarcopenia demonstrate various deteriorations in their physical performance [30,31]. Gait speed is a representative indicator of physical performance and is included in the diagnostic criteria for sarcopenia [6,7]. Diagnostic criteria commonly use the results of a 4- or 6-m *usual gait speed* test. Analyzing associations between daily life gait speed, muscle strength, and muscle mass, daily life gait speed also accurately reflects the sarcopenic state.

Advances in wearable devices have made it possible to continuously measure daily gait speed in the real world. As men usually wear belts in their daily lives, belt-type wearable devices can be used to measure daily life gait speed as naturally as possible over a long period. In fact, smart belt wearers can check their average walking speed on a daily, weekly, or monthly basis using a specific application. Therefore, older people would be able to identify a decrease in their physical performance in real life and, if so, would be able to visit health care providers for active intervention.

### Strengths and Limitations

This study had several strengths. First, the analysis was performed using gait speed data measured more than 200,000 times over a period of ≥10 days. Therefore, the results reflect the actual gait speed of the participants more accurately than the current common practice of *usual gait speed* measured in a laboratory at only one point in time. Second, the skeletal muscle mass of participants was accurately measured by the standard method using DEXA, and it was confirmed that the skeletal muscle mass of the lower limbs had a statistically significant association with gait speed. Third, we found that walking speed could be measured continuously over a long period using a belt-type wearable device. On the basis of these results, it is feasible to carry out more precise longitudinal studies in the future, including studies focused on the prediction of a negative prognosis related to a change in walking speed.

This study has some limitations. First, we could not determine a causal relationship between skeletal muscle mass and gait speed because of the limitations inherent to the cross-sectional study design. A second limitation was that the study included only male participants. We chose male participants as Korean women who met our age criterion of >50 years do not usually wear belts, and we wanted to be certain that the participants could wear the device with ease. Accordingly, the findings observed in this study cannot be applied to women who usually do not use belts. To overcome this problem, we considered two solutions to this limitation. One was to improve the esthetic look of the belt so that women could wear it as a fashion accessory. The other was to place a plastic case containing the sensor, circuit, and battery in the same position as the buckle of the belt in the form of a clip.

Finally, we developed an algorithm for detecting and analyzing gait speed, but this has not been fully validated. However, a previous study reported that a method based on the detection of heel-strike events using a triaxial accelerometer to confirm that each step is suitable for measuring gait speed [32]. In our unpublished data, when comparing the gait data measured using WELT with that measured using video readings in 10 healthy adults, the mean step count was 95.9% (SD 4.2%) consistent. The mean sensitivity of the WELT step detection algorithm was 87.9% (SD 2.8%), and the mean positive predictive value was 92.4% (SD 3.3%). However, there were no data regarding gait speed in patients with pathological gait patterns such as neurologic (Parkinson disease) or orthopedic conditions. Further studies are required to confirm the validity of wearable device–based gait speed measurements.

### Conclusions

Diverse and accurate information can be obtained by measuring daily life gait speed using a wearable device. Daily life gait speed is significantly associated with age and skeletal muscle mass of the lower limbs. As gait speed is a representative indicator of physical performance, older individuals would be able to detect a decrease in physical performance in real life by checking their walking speed. In addition, as it is possible to measure daily gait speed continuously over a long period using a wearable device, daily life gait speed data will be available for use in future longitudinal studies. Further studies are needed, especially studies aimed at developing a method to accurately measure women’s daily life gait speed as naturally as possible.

## Abbreviations

DEXA: dual-energy x-ray absorptiometry
WELT: wearable smart belt
